# Editorial: Untangle the broad connections and tight interactions between human microbiota and complex diseases through data-driven approaches, volume II

**DOI:** 10.3389/fmicb.2024.1449474

**Published:** 2024-07-10

**Authors:** Qi Zhao, Zuo-Bin Zhu, Jian Li, Li Zhang, Liang Wang

**Affiliations:** ^1^School of Computer Science and Software Engineering, University of Science and Technology Liaoning, Anshan, China; ^2^Department of Genetics, School of Life Sciences, Xuzhou Medical University, Xuzhou, China; ^3^School of Public Health and Tropical Medicine, Tulane University, New Orleans, LA, United States; ^4^School of Biotechnology and Biomolecular Sciences, University of New South Wales, Sydney, NSW, Australia; ^5^Laboratory Medicine, Guangdong Provincial People's Hospital, Guangdong Academy of Medical Sciences, Guangzhou, China; ^6^Department of Laboratory Medicine, Shengli Oilfield Central Hospital, Dongying, Shandong, China

**Keywords:** human microbiota, complex diseases, data-driven approaches, machine learning, artificial intelligence

The human microbiota is essential for maintaining health and shaping disease states, which encompasses trillions of microorganisms that reside in various body sites such as oral, skin, stomach, and intestine. The human microbiota is a dynamic ecosystem that is involved in essential functions such as digestion, immune modulation, and protection against pathogens, while the composition and function of these microbial communities are intricately linked to numerous complex diseases, including metabolic, inflammatory, and neurodegenerative disorders. However, due to the large volume of data generated from human microbiota analysis, it is difficult to conduct their analyses through traditional statistical methods. In contrast, advanced data-driven approaches such as bioinformatics and machine learning algorithms are needed to elucidate the compositional patterns and physiological functions of human microbiota in complex diseases, which have revolutionized our ability to unravel the connections between human microbiota and complex diseases, providing deeper insights into the microbiota-disease interplay. In recent years, there has been a growing use of machine learning (ML) and artificial intelligence (AI) techniques to analyze complex microbiome data. These techniques are superior at handling large datasets, identifying patterns, and making predictions that are not apparent through traditional statistical approaches. For example, random forests and support vector machines are representative supervised machine learning algorithms, which have been used to classify human disease states based on the corresponding microbiome profiles. Despite the progress, challenges remain in the fields such as inter-individual variability in microbiome composition, which further demonstrate the importance and necessity of data-driven approaches in the analysis of human microbiota data. Taken together, data-driven approaches have significantly facilitated the understanding of the human microbiota and its intricate connections to complex diseases, which can ultimately lead to improved diagnostics, treatments, and prevention strategies for complex diseases.

In this Research Topic, our goal was to emphasize the intriguing connection between human microbiota and complex diseases using data-driven approaches. These approaches allowed us to gain a deeper understanding of the interactions and mechanisms between human microbiota and complex diseases, providing new perspectives for disease prevention and treatment. In this Research Topic, we received a total of 58 submissions, of which 30 were accepted for publication after rigorous reviews.

Specifically, 13 published articles focused on the causal association between microbiota and complex diseases by a Mendelian randomization investigation. Yan W. et al. performed a two-sample Mendelian randomization study to investigate the associations between sleep disorders and specific components of the gut microbiota. Their findings revealed a significant causal relationship between four particular gut microbiota and sleep disorders. Su Q. et al. discovered possible causal links between 21 bacterial taxa and gastrointestinal cancers. Through Reverse Mendelian randomization analyses, they found that 17 microbial taxa were consistently associated with gastrointestinal cancer across all three Mendelian randomization (MR) methods. Their results may offer valuable biomarkers for the early, non-invasive detection of gastrointestinal cancer. The research conducted by Zhang J. et al. was the first to explore the causal relationship between the gut microbiome and the risk of primary biliary cholangitis (PBC). Their findings indicated that *Ruminococcaceae* and *Peptostreptococcaceae* were negatively associated with the risk of PBC. Conversely, *Selenomonadales, Bifidobacteriales*, and *Lachnospiraceae_UCG_004* appeared to have a potentially adverse effect on PBC. In another work, Zhang X. et al. examined the causal relationship between gut microbiota and hypothyroidism, investigating how specific microbial taxa influence the condition. They discovered that *Defluviitaleaceae/Defluviitaleaceae_UCG_011* acted as protective factors against hypothyroidism, whereas *Bacteroidaceae/Bacteroides* were identified as risk factors. Additionally, *Defluviitaleaceae* might prevent hypothyroidism triggered by subacute thyroiditis through interactions with influenza. Chronic renal failure (CRF), on the other hand, signifies a more advanced stage of kidney disease. Liu et al. concentrated on the later stages of CRF, and evaluated the genetic-level causal relationship between the gut microbiota of 119 genera and CRF using large-scale GWAS data. Through Mendelian randomization analysis and false discovery rate correction, they identified *Escherichia-Shigella* and *Howardella* as potential risk factors for CRF. Guo et al. employed the summary statistics from the MiBioGen consortium GWAS meta-analysis on the gut microbiome and from the FinnGen consortium on immune thrombocytopenia (ITP) to perform a two-sample Mendelian randomization analysis. Their analysis revealed that 10 gut microbial taxa might influence the risk of developing ITP. In another study, Wang Q. et al. provided the first direct evidence of a causal relationship between anti-H. *pylori* IgG levels and Myocardial Infarction (MI) using MR analysis. They conducted that higher anti-*H. pylori* IgG levels are associated with increased risks of MI in the European population, possibly due to lower HDL cholesterol levels. Similarly, The two-sample MR study carried out by Zhang T. et al. highlighted the significant role of gut microbiota and serum metabolites in the risk of intracerebral hemorrhage (ICH), identifying *Eubacterium xylanophilum*, genus *Senegalimassilia*, and isovalerate as strong causal factors. Qin et al. conducted a two-step MR analysis to investigate the mediating role of metabolic factors in the link between gut microbiota and cerebrovascular disease, finding both beneficial and detrimental causal effects of gut microbiota composition on cerebrovascular disease. Xu X. et al. used comprehensive GWAS summary statistics to explore the potential causal relationship between gut microbiome/metabolite levels and psoriatic arthritis risk, identifying Genus *Odoribacter* and Family *Rikenellaceae*, along with five peptide-related metabolites, as significant factors. Fu et al. employed a bidirectional two-sample MR analytical method with data from GWAS databases on gut microbiota and androgenetic alopecia (AGA), revealing significant insights into how gut microbiota influences AGA. Shen et al. established a causal link between gut microbiota and the hemorrhagic stroke through univariate and multivariate MR analyses, finding that *Dorea (genus), Eisenbergiella (genus)*, and *Lachnospiraceae* UCG008 *(genus)* may increase hemorrhagic stroke risk, while Rikenellaceae RC9 gut group *(genus)* and *Actinobacteria (phylum)* may decrease it. Xu R. et al. rigorously screened single nucleotide polymorphisms in GWAS summary statistics for gut microbiota and osteomyelitis, confirming a causal relationship through MR analysis and identifying one taxon of significant importance and six taxa of nominal significance.

Six articles focused on developing/evaluating computational models in microbiota related to complex diseases. Hu S. et al. introduced a method that combines the docking information with site-specific phage display to obtain a single chain variable fragment (scFv) with high affinity to salbutamol (SAL). They successfully identified highly efficient Anti-SAL scFv antibodies. Additionally, they fused the selected scFv with alkaline phosphatase and expressed in *E coli* to develop a rapid and low-cost one-step ELISA for detecting SAL. Chen Z. et al. presented a novel computational model called MLFLHMDA, which uses a multi-view latent feature learning approach to predict potential human microbe-disease associations. In global LOOCV and 5-fold cross-validation, MLFLHMDA achieved AUC scores of 0.9165 and 0.8942, respectively, outperforming six other methods. Hu H. et al. proposed a deep learning framework named the single-cell virus detection network (scVDN) to predict the infection status of single cells. Their results showed that scVDN outperforms four state-of-the-art machine learning models in identifying SARS-CoV-2-infected cells, even with highly imbalanced labels in real data. In another work, Qu et al. developed an ensemble learning model called MHBVDA to predict virus–drug associations using matrix decomposition with heterogeneous graph inference and bounded nuclear norm regularization. MHBVDA demonstrated superior performance compared to other models based on cross-validation result. Case studies involving ZIKV, SARS-CoV-2, HIV-1, and *Pseudomonas aeruginosa* further confirmed MHBVDA's excellent prediction capabilities. Peng et al. created a computational method named GPUDMDA to predict microbe–disease association by combining graph attention autoencoder, positive-unlabeled learning, and deep neural networks, using GPUDMDA, they identified potential associations between *Enterobacter hormaechei* and both asthma and inflammatory bowel disease, warranting further biological validation. Liao et al. proposed a model named MTCL-MDA based on graph collaborative filtering, which accurately predicts miRNA-disease associations. Comparative results with the existing methods demonstrate the superior performance of MTCL-MDA.

The remaining 11 articles involve other aspects of microbiota. For example, Wang X. et al. employed 2bRAD sequencing to accurately characterize the low biomass microbiome at species-level resolution. Their meta-analysis found increased levels of *Chlamydophila_abortus* and *CAG-873_sp900550395* in the ovarian cancer tissues, while several other species, such as *Lawsonella_clevelandensis_A, Ralstonia_sp001078575, Brevundimonas_aurantiaca, Ralstonia_sp900115545, Ralstonia_pickettii, Corynebacterium_kefirresidentii, Corynebacterium_sp000478175, Brevibacillus_D_fluminis, Ralstonia_sp000620465*, and *Ralstonia_mannitolilytica* were more abundant in the benign ovarian tissues. Li N. et al. constructed rat models for noise-induced hearing loss using different noise exposure levels. They found significant correlations between differential flora and metabolites during noise exposure, providing insights for preventing and controlling noise pollution. Jiang et al. conducted a two-month intervention study among Chinese adults to investigate the relationship between the gut microbiome and selenium absorption. They discovered that selenium supplementation had minimal impact on overall gut microbiome diversity but was linked to specific subsets of microorganisms. Yang X-T. et al. performed proteomic and metaproteomic analysis to explore the microbial features in gallstone and gallstone-free bile samples. Their findings shed light on the dysbiosis of resident microbes and the molecular interaction between the microbiome and the host, contributing to the understanding of gallstone formation. Li D. et al. studied the protective effects of glucagon-like peptide-2 (GLP-2) on dextran sodium sulfate-induced ulcerative colitis in mice. Their integrative analysis showed that GLP-2 inhibits the NF-κB and JAK/STAT3 inflammatory pathways, regulates glucose metabolism, and enhances intestinal protection by increasing dominant strains and regulating flora diversity. Yan P. et al. used 16 s rDNA and whole metagenome sequencing to analyze the gut microbial profiles in heroin use disorder (HUD) patients undergoing addiction, withdrawal (compulsory detoxification), and methadone maintenance treatment. They found significant associations between the presence of *Actinomyces, Turicibacter*, and *Weissella* and Hamilton Depression Scale scores. Chen X. et al. reviewed advancements in magnetic resonance imaging (MRI) technology, emphasizing its role in the early detection and identification of complex pathological changes in tuberculous meningitis (TBM). They highlighted the enhanced impact of integrating artificial intelligence into MRI for TBM diagnostics. In another review work, Shi et al. scrutinized the efficacy of emerging technologies, like machine learning, in transforming TBM diagnostics and management. They discussed advanced diagnostic tools such as targeted gene sequencing, real-time polymerase chain reaction (RT-PCR), miRNA assays, and metagenomic next-generation sequencing (mNGS) for early TBM detection. Guo and Zhang discussed the diverse mechanisms by which the gut microbiota is related to endometriosis, suggesting new avenues for prevention, early diagnosis, and treatment of the disease. Su Y. et al. used data from the cancer genome atlas (TCGA) database to compare microbial composition differences between 267 patients with early and 224 patients with advanced lung adenocarcinoma. They identified microbial markers related to cancer stage, demonstrating how the microbiome can complement other omics information and assist in clinical diagnosis. Yang M. et al. demonstrated that irisin treatment alone can alter intestinal flora and metabolite composition, indicating that intestinal flora is a target of irisin regulation. This finding suggests that irisin may mimic the effects of exercise in treating polycystic ovary syndrome.

In summary, we extend our heartfelt thanks to all the authors who contributed their original research to our Research Topic and to the reviewers for their insightful comments. We are also deeply grateful to the editorial office of Frontiers in Microbiology for their outstanding support and for giving us the opportunity to successfully present this hot Research Topic.

## Author contributions

QZ: Investigation, Methodology, Writing – original draft. Z-BZ: Writing – review & editing. JL: Writing – review & editing. LZ: Writing – review & editing. LW: Investigation, Methodology, Writing – original draft.

